# The degeneration changes of basal forebrain are associated with prospective memory impairment in patients with Wilson's disease

**DOI:** 10.1002/brb3.2239

**Published:** 2021-06-14

**Authors:** Yutong Wu, Sheng Hu, Yi Wang, Ting Dong, Hongli Wu, Yumei Zhang, Qianqian Qu, Anqin Wang, Yinfeng Yang, Chuanfu Li, Hongxing Kan

**Affiliations:** ^1^ School of Medical Information Engineering Anhui University of Chinese Medicine Hefei Anhui China; ^2^ Medical Imaging Center The First Affiliated Hospital of Anhui University of Chinese Medicine Hefei Anhui China; ^3^ Centers for Biomedical Engineering University of Science and Technology of China Hefei Anhui 230027 China

**Keywords:** basal forebrain, functional connectivity, functional magnetic resonance imaging, prospective memory, voxel‐based morphometry, Wilson's disease

## Abstract

**Introduction:**

Degeneration changes of the basal forebrain (BF) are suggested to play an important role in cognitive impairment and memory loss in patients with Alzheimer's disease and Parkinson's disease. However, little is known about if and how the structure and function of BF are abnormal in Wilson's disease (WD).

**Methods:**

Here, we employed the structural and resting‐state functional magnetic resonance imaging (fMRI) data from 19 WD individuals and 24 healthy controls (HC). Voxel‐based morphometry (VBM) and functional connectivity analysis were applied to investigate the structural and functional degeneration changes of BF in WD. Moreover, the linear regression analyses were performed in the patient group to depict the correlations between the aberrant gray volume and functional connectivity of the BF and clinical performances, such as the prospective memory (PM) and mini‐mental state examination (MMSE).

**Results:**

VBM analysis showed that compared with HC, the volume of overlapping cell groups of BF termed CH1–3 and CH4 was significantly reduced in WD. Additionally, the decreased functional connectivity of the CH4 was distributed in the bilateral temporal‐parietal junction (TPJ), right thalamus, orbitofrontal gyrus (ORB), and left middle cingulate cortex (MCC). The performances of the time‐based prospective memory (TBPM) and event‐based prospective memory (EBPM) were related to reduced functional connectivity between CH4 and right ORB. Besides, the functional connectivity of left TPJ was also significantly correlated with EBPM in WD.

**Conclusion:**

These findings indicated that the degenerative changes of BF may affect PM through the innervation brain function and may help to understand the neural mechanisms underlying cognitive impairment in WD.

## Graphical abstract

Voxel‐based morphometry (VBM) and functional connectivity analysis based on RS‐fMRI were applied to investigate the structural and functional degeneration changes of basal forebrain in Wilson's disease. The linear regression analyses were performed to depict the correlations between the aberrant functional connectivity of the basal forebrain and clinical variables, such as the prospective memory (PM) and Mini‐mental State Examination (MMSE) performances. Degeneration changes of basal forebrain affect PM by innervated brain function and may help to understand the neural mechanisms underlying cognitive impairment in WD.

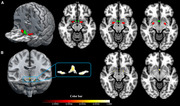

## 1 INTRODUCTION

Wilson's disease (WD) is an inborn disorder of copper metabolism, caused by excessive copper accumulation in the liver and brain (Huster, 2010). The main clinical symptoms include abnormal liver function, liver cirrhosis, tremor, chorea, dystonia, dysphagia, cognitive impairment, and Kayser‐Fleischer (KF) ring (Ala et al., 2007 ). Recent studies have reported that cognitive impairment mainly affects executive functions involving fronto‐striatal circuits (working memory, inhibitory process, and abstract reasoning) in WD patients (Wenisch et al., 2013). However, these studies did not provide direct neuroimaging features to characterize cognitive dysfunction in WD patients. Furthermore, cognitive impairment can be easily masked by other clinical symptoms, such as subcortical dementia and emotional dysfunction (Lang, 1989; Lang et al., 1990; Lorincz, 2010 ). Therefore, it is important to find the neuroimaging features of cognitive function to assist clinical diagnosis in patients with WD.

The basal forebrain (BF), containing four overlapping cell groups (CH1–CH4), plays a crucial role in the production of neurotransmitters to the neocortex, amygdala, and hippocampus (Mesulam & Geula, 1988; Mesulam et al., 1983 ) and also involves in the regulation of neuronal excitability and distinct cognitive function. Previous studies have reported that cognitive function decline is due to the significant degeneration of BF neurons and the loss of cortical cholinergic innervation (Mesulam, 2004). Literature review advance to find that BF cholinergic neurons are vulnerable to degeneration in Alzheimer's disease (AD) and Parkinson's disease (PD), and the neuronal loss in the BF is associated with the degree of cognitive function decline in AD and PD (Gargouri et al., 2019; Mesulam, 2004 ). Subcortical nuclei are also vulnerable to degeneration in WD. Hence, it is reasonable to hypothesize that BF neurons are vulnerable to degeneration, and its degeneration is associated with cognitive decline in WD.

Memory is an important component of cognitive function and is closely related to living standards and daily activities (Jing et al., 2019; Kim et al., 2006 ). Prospective memory (PM), the main component of memory, is defined as the plans or intentions of memory (Burgess et al., 2001; Okuda et al., 2007). Previous research divided PM into time‐based expected memory (TBPM), which refers to the memory for performing an operation at a specified time, and event‐based expected memory (EBPM), which refers to the memory for performing an operation when the target event occurs (Burgess et al., 2001; Okuda et al., 2007). Previous studies have shown that different parts of the central nervous system (including brainstem, cerebellum, thalamus, and subcortical white matter) can be affected in the case of WD, but are most common in basal ganglia (Seniow et al., 2002; Starosta‐Rubinstein et al., 1987; van Wassenaer‐van Hall et al., 1996; Zhong et al., 2019 ). Studies have shown that patients with basal ganglia pathology show significant memory impairment (Portala et al., 2001). It has been revealed that PM damaged in WD patients is associated with volume reductions of basal ganglia and abnormities in white matter fibers (Dong et al., 2016; Gargouri et al., 2019 ). However, the relationships between PM impairment and BF degeneration in patients with WD remain largely unknown.

In this study, we hypothesize that the BF neurons degenerate in WD, and its innervated functional network would be disrupted, which is associated with PM impairment in WD patients. To prove this hypothesis, we adopted voxel‐based morphometry (VBM) analysis to evaluate the differences in BF gray volume between WD and healthy controls (HC) and then selected atrophic BF areas as a region of interest (ROI) for functional connectivity analysis. To examine the effect of the BF on the memory performance in patients with WD, we performed linear regression analysis in the patient group to explore whether the abnormal structure and functional connectivity (FC) of the BF were correlated with clinical symptoms.

## 2 MATERIALS AND METHODS

### 2.1 Participants

A total 19 patients with neurological WD and 24 healthy controls (HC) matched with age and sex, who had been recruited from the First Affiliated Hospital of Anhui University of Chinese Medicine (AUCM). The HC group included 11 women (45.8%) with a mean age of 22.78 years (SD = 7.34 years). The WD group included eight women (42.1%) with a mean age of 22.39 years (SD = 6.35 years). All patients were receiving drug treatment, including penicillamine and zinc salts. Comprehensive clinical interviews were evaluated by experienced neurologists and diagnosis of WD based on clinical symptoms, including presence of KF ring, neuroimaging findings, and abnormal copper metabolism. None of the patients had a history of neurological diseases other than WD. Healthy controls had no history of head injury, neurological disorder, or concomitant medical disorder. All participants provided written informed consent. This study has been carried out in accordance with the Code of Ethics of the World Medical Association (Declaration of Helsinki) and received ethical approval from the Human Research Committee of the First Affiliated Hospital of AUCM. More details about clinical and demographic characteristics are shown in Table 1.

**TABLE 1:** Clinical features of patients and healthy controls

	WD (*N* = 19)	HC (*N* = 24)	*p* value
Gender (male/female)	11/8	13/11	.833
Age (years)	10–36)23±6.35(	17–27)23±7.34(	.890
Education (years)	7–16)9.16±2.12(	7–16)9.29±2.40(	1.000
Handedness	19 right‐handed	24 right‐handed	
WD duration (years)	1–10)5.45±3.14(	—	
WD types	Neurological	—	
The KF ring	27 WD with the KF ring	—	
24‐h urinary Cu (μmol/day)	1−6 (2.32 ± 1.49)	—	
CP (mg/dl)	1−9(5.3 ± 2.53) mg/dL	—	
MMSE	25–28)26.42±0.90(	—	
TBPM	1–6)2.95±1.68(	—	
EBPM	2–8)4.74–1.52(	—	

Abbreviations: CP, ceruloplasmin; Cu, copper; EBPM, event‐based expected memory; HC, healthy controls; KF, Kayser–Fleischer; MMSE, mini‐mental status examination; N, number; TBPM, time‐based expected memory; WD, Wilson's disease.

### 2.2 Neuropsychological evaluation

In our study, the comprehensive neuropsychological assessments of WD patients were examined by an experienced neuropsychologist, including (1) mini‐mental state examination (MMSE) which measured temporal and spatial orientation, memory, language, and visuospatial skills, and (2) PM which contains EBPM and TBPM. PM test was performed as mentioned in previous studies (Esposito et al., 2015; Gonneaud et al., 2014; Loprinzi et al., 2018).

The EBPM test initially required the subjects to tap the desk whenever they found two animal words (task events) during the task. They were asked to provide their phone number after the tests were finished. Next, 30 question cards were provided for the subjects to perform the word selection task. Each card was printed with 12 Chinese words. Ten of 12 words belonged to one category and the remaining two words belonged to another category. The experimenter showed each card to the subjects who were asked to choose two words that belonged to a category that differs from the other 10 words, and then asked to spell these out at their own pace. According to the instructions before the experiment, the EBPM task of subjects was to tap the desk when they met the target word (animal word), and one target event was done. Target events occurred on the 5th, 10th, 15th, 20th, 25th, and 30th card of the word selection task. One point was awarded when the subjects correctly responded to a target event (a total of six target events). Two points were awarded when subjects remembered to provide the telephone number after the test. No points were awarded when subjects incorrectly responded to a target event or forgot to provide their phone number after the test. The maximum score in the EBPM task was 8.

The TBPM test instructed the subjects to tap the desk at 5‐min intervals from the start time. During the test, subjects were allowed to use a digital clock to check the time. The clock was placed one meter behind the subject's right shoulder to eliminate any visible prompts. The clock was set to 00:00:00 at the beginning of the testing. After the clock was started, the subjects were instructed to perform the number selection task. The task contained 100 cards, each of which was printed with 12 two‐digital numbers. The subjects were instructed to select the largest and smallest number in the cards. The exact time was recorded when the subject responded by tapping the desk. The number selection task was stopped when the clock indicated 17 minutes. Two points were awarded if the subjects responded from 10 s before to 10 s after the target time. One point was awarded if the subjects responded from 30 s before to 30 s after the target time. The maximum score of the TBPM was 6. The neuropsychological data were summarized in Table 1.

### 2.3 Data acquisition

All data of healthy controls and patients were acquired at the First Affiliated Hospital of AUCM, by using a 3.0 Tesla MRI scanner (Discovery MR750, General Electric) with a standard eight‐channel head coil for signal reception. All participants were instructed to rest with their eyes closed but not to fall asleep during scanning. Special soundproof earplugs and foam pads were used to avoid noise‐induced discomfort and head motion. The MRI protocol was as follows: Sagittal T1WI high‐resolution images were collected by using T1‐3D BRAVO sequence with the following acquisition parameters: repetition time (TR) = 8.16 ms, echo time (TE) = 3.18 milliseconds, flip angle (FA) = 12 degrees, field of view (FOV) = 256 mm × 256 mm, matrix = 256 mm × 256 mm, 200 axial slices with 1 mm thickness; resting‐state functional MRI images were acquired by using gradient‐echo single‐shot echo‐planar imaging sequences with a TR = 2000 ms, TE = 30 ms, FOV = 220 mm × 220 mm, FA = 90°, matrix = 64 × 64, slice thickness = 3 mm, with 185 volumes. For data quality control, the scan was evaluated by two experienced neuroradiologists who were blind to clinical information.

### 2.4 MRI analysis

#### 2.4.1 Resting‐state fMRI data preprocessing

All the resting‐state fMRI images were preprocessed by using Analysis of Functional NeuroImage (AFNI, Version: 19.2.21, http://afni.nimh.nih.gov/afni/) software. The first ten volumes of each participant were discarded to eliminate transients and account for T1 relaxation effects, followed by slice timing correction to compensate for the time delay across slices. Motion correction was performed by realigning all functional images to the middle image and the data with head motion over 2 mm or 2° were excluded. Then, the motion‐corrected functional volumes were co‐registered to the high‐resolution anatomical images, followed by normalizing functional images to the Montreal Neurological Institute (MNI) standard brain. All fMRI data were spatially smoothed with a Gaussian kernel of 6‐mm full‐width at half maximum (FWHM). The data were linearly detrended, and the residual signals were band‐pass temporal filtered at 0.008–0.1 Hz to reduce low‐frequency drifts and high‐frequency noise.

#### 2.4.2 Definition of the BF

**FIGURE 1:**
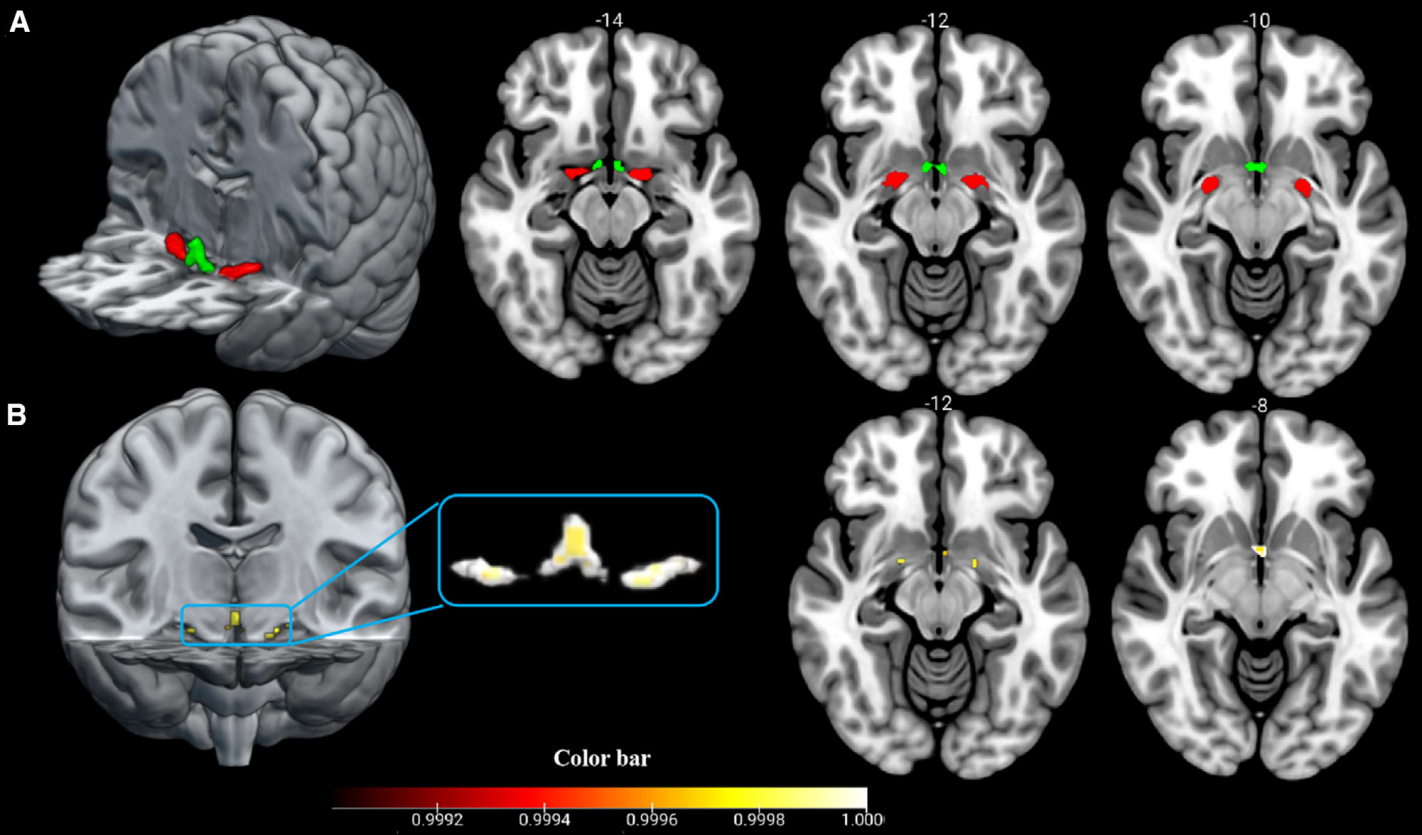
MR image shows BF seed regions and its group differences in volume. (a) The seed regions of BF, including CH1–3 (green) and CH4 (red), were displayed on the axial slice and 3D stereogram in the Montreal Neurological Institute (MNI) space. (b) The volume of CH1–3 and CH4 were significantly reduced in WD patients. The color bar represents *p* value and 1 – *p* represents the significant differences between two groups

We define the BF by using Anatomy toolbox r 3.0 toolbox (Eickhoff et al., 2005) (https://github.com/inm7/jubrain‐anatomy‐toolbox) in conjunction with the Statistical Parametric Mapping (SPM, Version 12, https://www.fil.ion.ucl.ac.uk/spm) in Matrix laboratory (Release 2016a, MathWorks, Natick, MA, USA). The CH1–3 and CH4 were defined using probability maps of BF which are contained in the Anatomy toolbox. The ROIs were thresholded at 50% probability and then resampled and warped to the MNI standard brain (Figure 1a).

#### 2.4.3 BF volume estimation

BF volume measurements were performed using FSL‐VBM. The FSL (version 5.09, http://fsl.fmrib.ox.ac.uk/fsl/fslwiki/) Automated Segmentation Tool (FAST) was employed to segment gray matter (GM) from the high‐resolution anatomical images. The segmented GM parietal volume images were then normalized to the MNI152 standard space by performing linear image registration with a nonlinear registration toolbox. The normalized images were further averaged and flipped along the *x*‐axis to create a study‐specific GM template. All native GM images were subsequently nonlinearly registered to the study‐specific template and modulated for contraction due to the nonlinear component of the transformation by dividing them by the Jacobian of the warp field. The modulated GM images were finally smoothed using an isotropic Gaussian kernel with a sigma of 3 mm. The BF volume for each participant was extracted based on defined BF mask. The extracted BF volumes were further extracted to do statistical analysis.

#### 2.4.4 Functional connectivity analysis

Functional connectivity of CH1–3 and CH4 were calculated, respectively. First, the cerebrospinal fluid (CSF), white matter, and the signal of ROIs (CH1–3 and CH4) were extracted. Then, several sources of variance were removed from the data by linear regression as follows: (1) six parameters obtained by correction of head motion; (2) the signals from CSF; (3) the signals from WM. For each participant, the Pearson correlation coefficients between the mean time series of ROIs and the time series of every voxel across the whole brain were calculated and converted to a *z*‐value using Fisher *r*‐to‐*z* transformation to improve the normality.

### 2.5 Statistical analysis

Group difference in the gray volume of BF was assessed using permutation‐based non‐parametric testing with 5000 random permutations, which was performed by Randomise, a subcommand from FSL. The significance threshold was set at *p* < .001, using the threshold‐free cluster enhancement method with family wise‐error (FWE) correction for multiple comparisons. The relations between the volumes of BF and neuropsychological symptoms were measured by linear regression analysis and the results were corrected by FDR for multiple comparisons.

A group‐level analysis was applied to identify the group differences of FC by using 3dttest++, a subcommand from AFNI. Group‐wise whole‐brain analysis of the belief‐photo contrasted with a voxel‐wise *p* threshold of .001 and cluster‐wise threshold of .01. The relations between FC of WD patients in brain regions of significant differences and neuropsychological symptoms were measured by linear regression analysis and the results were corrected by FDR for multiple comparisons.

In order to evaluate whether head motion has influences on the results, we also performed the correlation analysis between frame‐wise displacement (FD) and neuropsychological symptoms.

## 3 RESULTS

### 3.1 Group differences in BF volumes

The WD group showed significant volume reductions in CH1–3 and CH4 when compared with HC. No increased volumes were observed in BF when compared to HC (Figure 1b). The altered volumes of CH1–3 (Figure S1) and CH4 (Figure S2) have no correlations with neuropsychological symptoms.

### 3.2 Group differences in FC of BF

**FIGURE 2:**
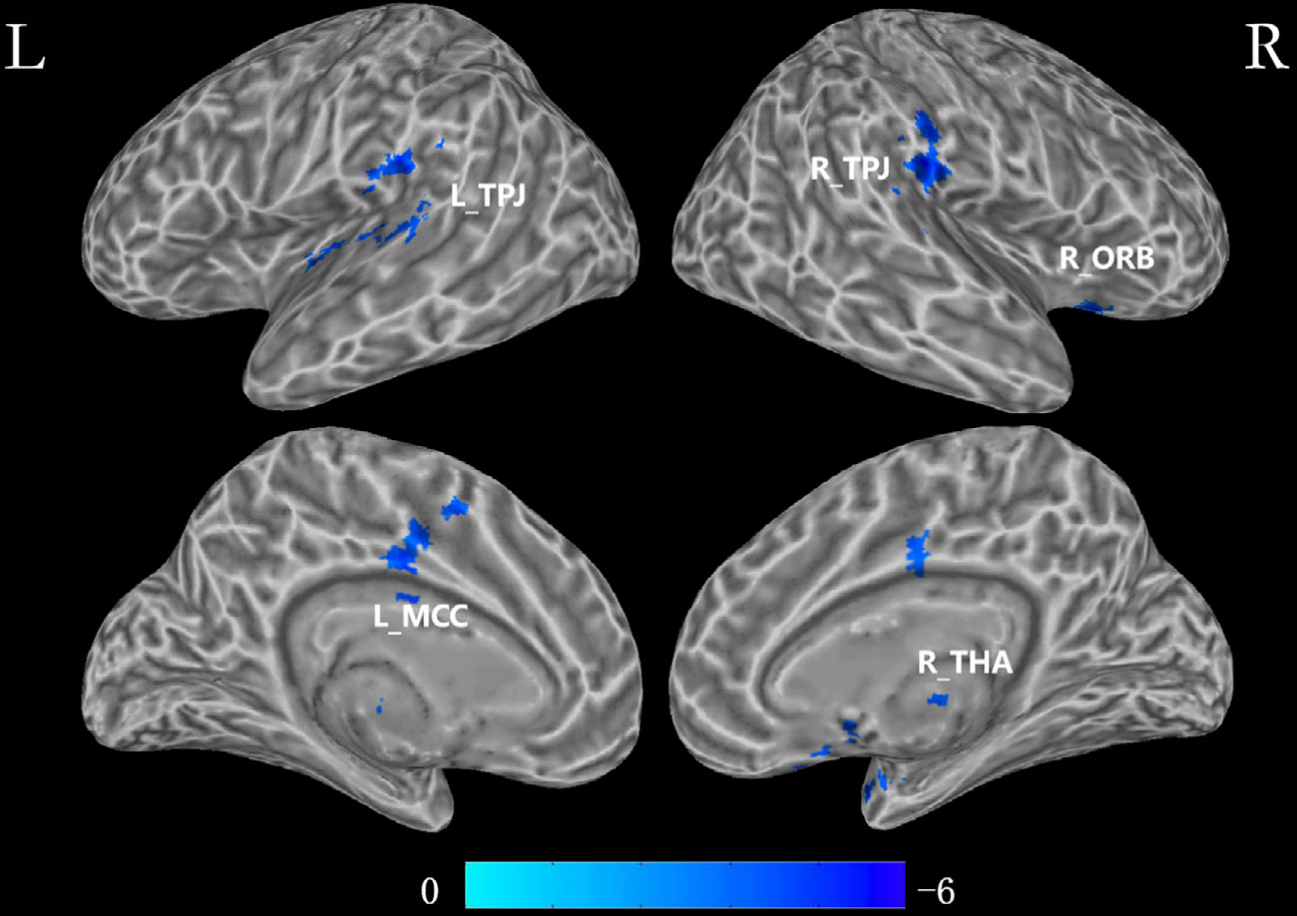
Group differences in functional connectivity of CH4. Group‐wise whole‐brain analysis of the FC in contrast with a voxel‐wise *p* threshold of .001 and cluster‐wise threshold of .01 Abbreviations: L, Left; L_MCC, left middle cingulate cortex; L_TPJ, left temporal‐parietal junction; R, Right; R_ORB, right orbitofrontal gyrus; R_TPJ, right temporal‐parietal junction; R_THA, right thalamus

**FIGURE 3:**
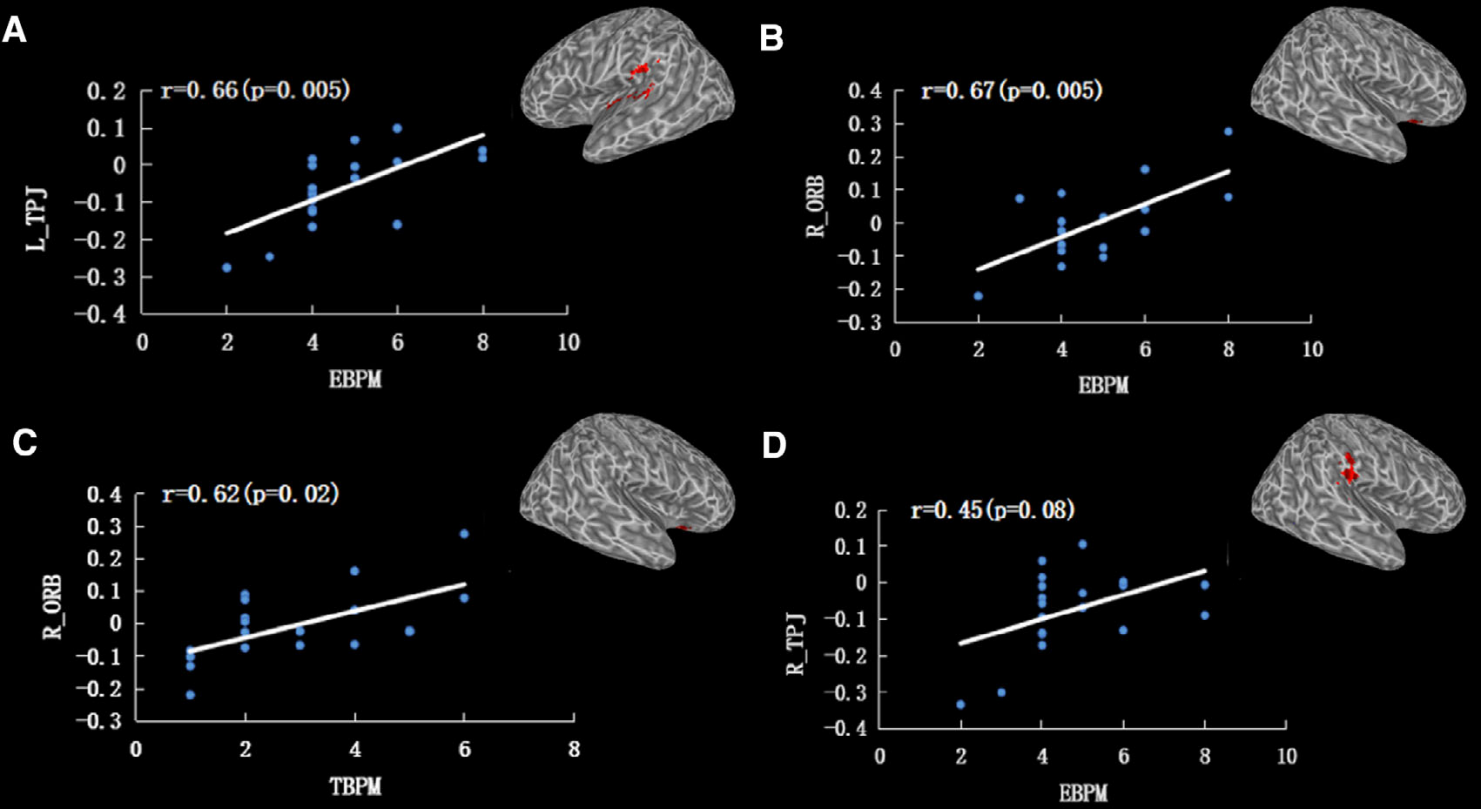
The correlations between PM and FC of CH4 in WD patients. (a) Correlation between L_TPJ FC and EBPM. (b) Correlation between R_ORB and EBPM. (c) Correlation between R_ORB FC and TBPM. (d) Correlation between R_TPJ FC and EBPM Abbreviations: EBPM, event‐based expected memory; L_TPJ, left temporal‐parietal junction; R_ORB, right orbitofrontal gyrus; R_TPJ, right temporal‐parietal junction; TBPM, time‐based expected memory

The WD patients showed reduced FC of the CH4, which was distributed in the bilateral temporal‐parietal junction (TPJ), right thalamus, right orbitofrontal gyrus (ORB), and left middle cingulate cortex (MCC) (Table 2 and Figure 2). A positive correlation (*r* = 0.62, *p* = .02) was observed between TBPM and the FC between CH4 and right ORB. FC in the right ORB (*r* = 0.67, *p* = .005) and left TPJ (*r* = 0.66, *p* = .005) also have positive correlations with EBPM (Figure 3).

**TABLE 2:** Group differences in functional connectivity of CH4

		MNI coordinate)mm(				
Region	Hemisphere	X	Y	Z	Voxel	*Z* value
TPJ	L	–62	–30	18	336	–4.56
TPJ	R	58	–26	20	333	–5.25
THA	R	8	0	0	237	–4.79
MCC	L	–2	–6	36	180	–4.22
ORB	R	28	16	–22	156	–4.61

*Note*: Group‐wise whole‐brain analysis of the FC in contrast with a voxel‐wise *p* threshold of .001 and cluster‐wise threshold of .01.

Abbreviations: L, Left; MCC, middle cingulate cortex; ORB, orbitofrontal gyrus; R, Right; THA, thalamus; TPJ, temporal‐parietal junction.

The head motion has no correlations with neuropsychological symptoms (Figure S3), and we have regressed out six parameters of head motion in FC analysis. Therefore, this indicated that group differences in FC and correlations between FC and neuropsychological symptoms were not influenced by head motion.

## 4 DISCUSSION

Examining the volume and function of the brain may be crucial for probing and understanding the mechanisms of many neuropsychiatric diseases. At present, the FC abnormalities caused by the degenerative changes of the BF in WD patients are still unclear. Hence, we systematically examined structural changes of BF and its functional connectivity changes in WD patients relative to HC. We used VBM analysis to investigate the BF atrophy and the atrophy BF was furtherly selected as ROIs to perform FC analysis based on resting‐state fMRI. Our results showed that the CH4 and CH1–3 were presented volume atrophy, and FC changes of CH4 were distributed in the bilateral TPJ, right thalamus, ORB, and left MCC. Furthermore, decreased FC between CH4 and right ORB was positively associated with TBPM and EBPM, and decreased FC between CH4 and left TBJ was also positively correlated with EBPM. Collectively, these findings suggest that degenerative change of CH4 may contribute to dysfunction, and furtherly has an effect on prospective memory in WD.

### 4.1 Structural changes in BF

The BF structures are located in the forebrain to be the front of and below of striatum. BF provides widespread cholinergic projection to the neocortex, which forms a part of the neuromodulation system that plays a significant role in cognitive function. In this paper, the WD group showed significant volume reductions in BF. Although there are no literature to report the degeneration changes of BF in WD patients, the BF is the structures that belongs to subcortical nucleus whose components have extensively neuronal loss and atrophy in WD, including putamen, caudate, pallidum, etc. (Lorincz, 2010; Page et al., 2004; Sinha et al., 2006). In other neurodegeneration disease, such as AD and mild cognitive impairment, previous study has found that the loss of memory function is due to the degeneration of CH4 neurons (Mesulam, 2004). In this study, volume atrophy of BF was not correlated with PM. But the degenerative changes of BF have influences on brain function, which is significantly associated with PM. This indicated that degeneration changes of BF may indirectly impact PM by affecting cortical function.

### 4.2 Functional connectivity changes in BF

The BF nuclei, containing four cell groups (CH1–CH4), serves as the major sources of cholinergic projection neurons to the neocortex, amygdala, and hippocampus (Mesulam & Geula, 1988; Mesulam et al., 1983; Woolf, 1991). Specifically, the CH4 sends the cholinergic projections to the neocortex and amygdala, whereas the hippocampus receives cholinergic inputs from the CH1–3. In this study, we found decreased FC between CH4 and bilateral TPJ in WD relative to HC. The TPJ, located in the region between the temporal and parietal lobe (Carter & Huettel, 2013), is involved in specifically social functions or nonspecific processes of cognition such as memory and attention (Carter & Huettel, 2013). Previous studies have found that structural and functional damage of TPJ is linked to behavioral and cognitive symptoms in disorders, such as amnesia (Sehm et al., 2011), AD (Salmon et al., 2005), autism spectrum disorder (Martinez‐Murcia et al., 2017; Pantelis et al., 2015 ), and schizophrenia (Das et al., 2012; Lee et al., 2011 ; Vercammen et al., 2010 ). In the present study, we found that FC between CH4 and left TPJ is positively correlated with EBPM. This indicates that such functional alterations might reflect a robust link between PM and cholinergic degeneration in WD.

Previous studies have well described the BF sends cholinergic projections to frontal cortical targets (Bloem et al., 2014; Chandler & Waterhouse, 2012; Chandler et al., 2013 ). Concretely, the medial frontal cortex receives projections from anterior BF (CH1–3), while posterior BF (CH4) sends the projections to the dorsal prefrontal cortical areas (Beckmann et al., 2005; Bloem et al., 2014; Damoiseaux et al., 2006). In our study, we observed that decreased FC of CH4 was distributed in the right ORB in WD patients when compared to HC. This functional change is positively correlated with the performances of the EBPM and TBPM, which further indicates that FC between CH4 and right ORB is a robust link between PM and cholinergic degeneration in WD. The decreased FC of CH4 was also observed in the left MCC and right thalamus. The cingulate cortex receives inputs from the thalamus and neocortex (Hayden & Platt, 2010; McFarland & Haber, 2000; Wyss & Van Groen, 1992), which is involved in emotion formation and learning (Fujiwara et al., 2007; Jing et al., 2019; Rolls, 2019; Wang et al., 2017). By the system literature review, no researches reported that the BF interactive with the thalamus and MCC are involved in memory process or cognitive function. FC of the left MCC and right thalamus were not related to the PM performances, which is expected to conclude that projections from CH4 to MCC and thalamus might not innervate PM or cognitive function in WD. The potential interpretation for FC decreased in the thalamus and MCC is that such FC changes are caused by other symptoms in patients with WD. Overall, these findings may indicate that the interaction between CH4 and the prefrontal cortex plays an important role in the administration of PM.

### 4.3 Limitations

Our research has several limitations that warrant discussion. First, healthy controls have not received the neuropsychological tests and were recruited based only on an unremarkable medical history, which may limit the neurophysiological alterations specific for individuals at risk for WD. Second, it may be difficult to define the BF and to eliminate the partial volume effect due to the small size of BF. Even with this limitation, findings in the WD cohort were consistent with other degenerative diseases. In the future, higher resolution fMRI, however, should be applied to further study the degenerative changes of BF in WD. Third, our work only focused on the BF to reveal how its degeneration affected PM, which limits understanding the results from the joint abnormal of BF and other regions. Further research should determine which other brain areas are related to memory impairment patients with WD.

## 5 CONCLUSIONS

In conclusion, we found that volume atrophy of BF in WD, as evaluated by brain MRI, is linked with brain function changes. Specifically, the significant FC changes of CH4 at the ORB are associated with TBPM and EBPM, and FC change at the left TPJ is positively correlated with EBPM. This study provides new evidence that the cholinergic BF degeneration is a crucial contributor to memory dysfunction in WD. Considering that the progress of WD is associated with clinical treatment, further longitudinal studies should be continued to investigate therapies that target the cholinergic BF.

## CONFLICT OF INTEREST

The authors declare no conflict of interest.

## AUTHOR CONTRIBUTIONS

Sheng Hu and Yutong Wu designed the experiment, analyzed experimental results, and wrote the manuscript; Yi Wang revised the manuscript; Hongli Wu and Ting Dong researched the literature and decided whether the literature was included when disagreement appeared; Anqin Wang, Yumei Zhang, Yinfeng Yang, and Qianqian Qu conducted preprocessing of MRI data; Hongxing Kan and Chuanfu Li guided, reviewed, and revised the manuscript and provided unique insights into the direction of the discussion.

### PEER REVIEW

The peer review history for this article is available at https://publons.com/publon/10.1002/brb3.2239.

## Supporting information



Supporting information

## Data Availability

The data that support the findings of this study are available from the corresponding author upon reasonable request.
