# Supplementary material for: The degeneration changes of basal forebrain are associated with prospective memory impairment in patients with Wilson's disease
Source: Brain Behav. 2021 Jun 14;11(8):e2239. doi: 10.1002/brb3.2239 (PMC8413803; doi:10.1002/brb3.2239)
Supplement: Supplementary file 1 — Supporting information [file BRB3-11-e2239-s001.doc]

**Supplementary information: Supplementary Figures 1-3.**


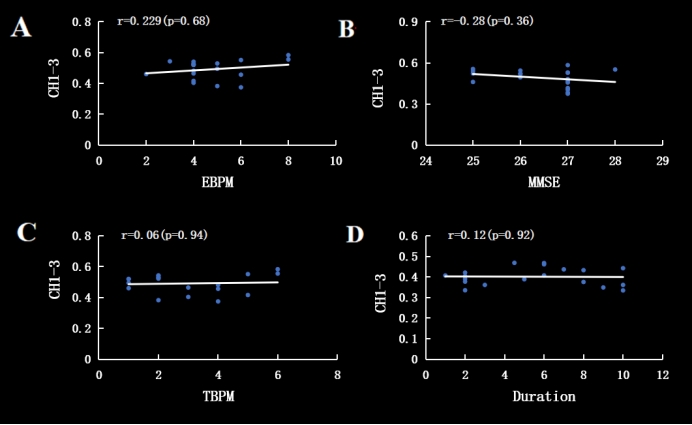


**Supplementary Figure 1.** **The correlations between clinical symptoms and volume of CH1-3 in WD patients.** A. Correlation between volume of CH1-3 and EBPM. B. Correlation between volume of CH1-3 and MMSE. C. Correlation between volume of CH1-3 and TBPM. D. Correlation between volume of CH1-3 and disease duration. Abbreviations: CH1-3, volume of CH1-3 in WD patient; MMSE, mini-mental status examination; TBPM, time-based expected memory; EBPM, event-based expected memory.


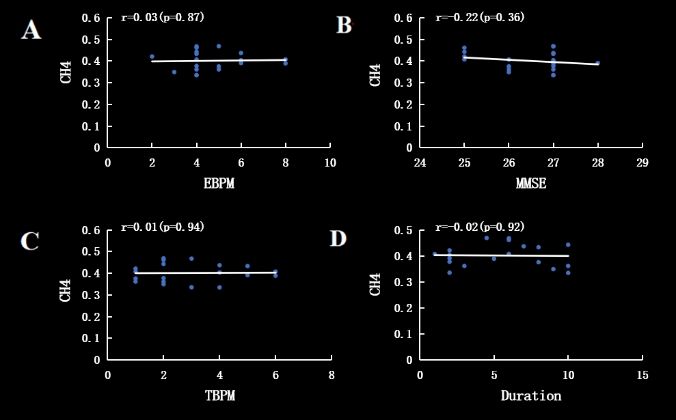


**Supplementary Figure 2. The correlations between clinical symptoms and volume of CH4 in WD patients.** A. Correlation between volume of CH4 and EBPM. B. Correlation between volume of CH4 and MMSE. C. Correlation between volume of CH4 and TBPM. D. Correlation between volume of CH4 and disease duration. Abbreviations: CH4, volume of CH4 in WD patient; MMSE, mini-mental status examination; TBPM, time-based expected memory; EBPM, event-based expected memory.


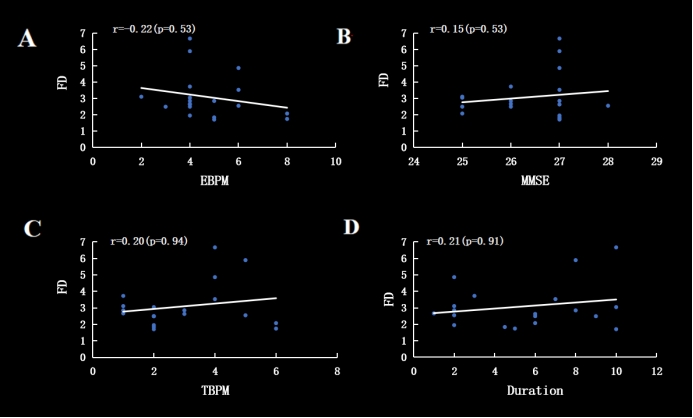


**Supplementary Figure 3.** **The correlations between clinical symptoms and head motion parameter in WD patients.** A. Correlation between FD and EBPM. B. Correlation between FD and MMSE. C. Correlation between FD and TBPM. D. Correlation between FD and disease duration. Abbreviations: FD, frame-wise displacement; MMSE, mini-mental status examination; TBPM, time-based expected memory; EBPM, event-based expected memory.
